# How Should Oncologists Choose an Electronic Patient-Reported Outcome System for Remote Monitoring of Patients With Cancer?

**DOI:** 10.2196/30549

**Published:** 2021-09-09

**Authors:** Fabrice Denis, Ivan Krakowski

**Affiliations:** 1 ELSAN Jean Bernard Institute Le Mans France; 2 Association Francophone pour les Soins Oncologiques de Support Bègles France

**Keywords:** ePRO, cancer, remote monitoring, quality, effectiveness, security, digital monitoring, digital health, cancer patients, patients with cancer, oncology

## Abstract

Electronic patient-reported outcome (ePRO) systems for symptom monitoring in patients with cancer have shown quality of life and survival benefits in controlled trials. They are beginning to be used in routine oncology practice. Many software developers provide software solutions for clinicians, but how should clinicians decide which system to use? We propose a synthesis of the main questions regarding the effectiveness, safety, and functionality of an ePRO system that a clinician should ask software providers to assist in the selection of a software product in order to obtain the best value tools for their patients and their practice.

## Background

Electronic patient-reported outcome (ePRO) systems for symptom monitoring of patients with cancer have had demonstrated quality of life and survival benefits in controlled trials (Table 1). They also result in a reduction of emergency hospitalization and have favorable cost-effectiveness and clinical utility; in addition, users have good perceptions of ePRO systems [1-9].

The practical integration of these ePRO systems into patient care is increasing and the positive results of trials have contributed to their increased use. The organization of medical teams is challenging because it requires the presence of a nurse dedicated to initial alert management and a dedicated time for the physician to respond to the alert.

It is challenging for clinicians and patients to identify systems that will add value to patient care in real life because the number of ePRO tools available is rapidly increasing, making it difficult for physicians to choose just one.

**Table 1:** Randomized studies of remote monitoring of patients with cancer by electronic patient-reported outcome systems.

Authors	Number of patients	Indications	Questionnaires used	Multicentric trial	Type of cancer	Improved outcome
Basch, 2017 [1] and 2016 [9]	766	Toxicity monitoring	NCI-CTCAE^a^	No	All	Quality of life, survival, reduced emergency use
Mir, 2020 [2]	609	Toxicity monitoring	PRO-CTCAE^b^	No	All	Dose intensity
Basch, 2020 [3]	1191	Toxicity monitoring, follow-up, supportive care	PRO-CTCAE	Yes	All	Quality of life, symptom control
Absolom, 2021 [4]	508	Toxicity monitoring	NCI-CTCAE	No	All	Quality of life, symptom control
Berry, 2014 [5]	752	Toxicity monitoring, supportive care	SDS-15^c^	Yes	All	Symptom control
Strasser, 2016 [6]	264	Toxicity monitoring, supportive care	ESAS^d^	Yes	All	Symptom control
Denis, 2019 [7]	133	Follow-up	Not reported	Yes	Lung	Survival
Moony, 2021 [8]	252	Toxicity monitoring	Not reported	No	All	Quality of life, symptom control

^a^NCI-CTCAE: National Cancer Institute version of the Common Terminology Criteria for Adverse Events.

^b^PRO-CTCAE: Patient-Reported Outcomes version of the Common Terminology Criteria for Adverse Events.

^c^SDS-15: Symptom Distress Scale-15.

^d^ESAS: Edmonton Symptom Assessment System.

Many criteria can be used to select ePRO systems, although there is little scientific literature focused on ePRO systems in oncology. However, some criteria on effectiveness, security, and functionality are usually included in telehealth or digital therapeutics assessments [10-12].

In general, ePRO systems must meet specific criteria for both the patient’s and clinician’s benefit. Here, we propose some questions that physicians may direct toward system providers to help them choose a relevant ePRO software.

## Effectiveness Criteria

1. Notifications must be sent to the medical team. This factor appears to be more important than sending a notification to the patient on their smartphone and allowing them to call the medical center. This was included in randomized studies of ePRO systems for patients with cancer [1-9].
2. The solution must be a medical device with quality marking such as a Class II CE mark [13-15]. This ensures that the product complies with the essential requirements of the relevant European Union legislation in which all devices must be evaluated for clinical efficacy and any side effects, if applicable, by means of preclinical and clinical evaluation. As ePRO systems are Class IIa medical devices, manufacturers must provide full quality assurance such as product and postmarket surveillance (materiovigilance) to obtain conformity. The management of materiovigilance is included in Class IIa CE marking and is mandatory.
3. The solution must use validated algorithms. This validation aims to assess reliability and the performance of algorithms in detecting events; algorithms’ performance should be published in journals [1-9,16,17].
4. The solution should contain algorithms that allow adverse events monitoring of the main drugs used in oncology (ie, chemotherapy, immunotherapy, hormonotherapy, targeted therapy, radiotherapy) [1-6,8,16,17].
5. As early supportive care improves survival in oncology, the solution should contain algorithms to detect symptoms that make a patient eligible for early supportive care [3,5,6,18].
6. The solution should allow for follow-up of the patient to detect complications or symptomatic relapse early, which improves clinical utility and patient outcomes [3,7].
7. Clinicians should use solutions with up-to-date algorithms, especially for patients receiving therapies such as maintenance or long-course treatment. As the main new drugs (eg, immunotherapy or targeted therapies) have only been widely used for fewer than 5 years, solutions using algorithms older than this should be avoided. This is mandatory for Class IIa CE marking of medical devices [13-15].
8. Validated questionnaires such as the Patient-Reported Outcomes version of the Common Terminology Criteria for Adverse Events (PRO-CTCAE) items from the National Cancer Institute or the Edmonton Symptom Assessment System (ESAS) should be used in the patient questionnaire. The PRO-CTCAE measurement system was developed to capture symptomatic adverse events in patients in cancer clinical trials and is now recommended for ePRO remote monitoring of symptoms because it evaluates the symptom attributes of frequency, severity, and interference. It has also been linguistically validated in more than 30 languages [3,19]. ESAS is one of the most used patient-reported outcome scales for symptom assessment in palliative care and oncology in the past 25 years. Although it only uses unidimensional scales to assess symptom intensity, ESAS has been psychometrically validated, has been translated into numerous languages, and is freely available. A change of one point was found to be the optimal cutoff for both improvement and deterioration for all 10 symptoms included in ESAS using a sensitivity-specificity approach. As it enables rapid, pragmatic assessment of multiple symptoms simultaneously, ESAS is used extensively in the clinical setting for symptom screening and monitoring worldwide. However, it cannot be used to monitor all treatment toxicities because only 10 symptoms are assessed; the ESAS does not include digestive symptoms (eg, vomiting, diarrhea), cutaneous symptoms, fluid retention (eg, edema), or sepsis. However, its routine use was associated with a 6% increase in 1-year overall survival in a retrospective matched cohort study of 128,893 patients [20-22].

## Safety Criteria

1. A real-life study assessment of the solution should be done to confirm that performance and compliance are in line with that reported in premarket studies and to assess the postmarket security of the solution. It is especially important to assess the satisfaction of patients in a real-life study [13].
2. In Europe, the solution must comply with the General Data Protection Regulation (GDPR) that protects and secures patient data. Data protection regulations strengthened the management of alerts and the security of data transmitted to the medical team [23].
3. Oncologists should avoid a solution that allows clinicians to set alert thresholds themselves because the lack of data about the safety and reliability of modified thresholds can result in physician responsibility if a patient experiences an adverse event (eg, following a false negative event).
4. Solutions that allow the creation of “homemade” algorithms should be avoided for the same reasons as described in the previous point.
5. Oncologists should avoid solutions developed by software providers about which a critical security alert was issued by health authorities (eg, risk of a serious adverse event due to critical software dysfunction). Many countries are already publishing these notifications on their health agency’s portal (eg, the French national health security agency [24]).

## Functionality

1. As not all patients have smartphones, the software should allow for the sending of forms by mail or SMS text message to patients [3].
2. The software must allow different health professionals (physicians and nurses and their colleagues) to manage notifications [3].
3. There should be one software for all major cancers, covering the entire course of treatment (active treatment and surveillance) to avoid the use of multiple software for one patient. This would reduce the technical burden placed on professionals and result in higher levels of satisfaction among patients and health professionals in routine use [3].
4. A single software containing an all-in-one algorithm enabling the detection of toxicities, follow-up management, and supportive care should be prioritized to avoid the use of multiple software with different algorithms. Too many different rules triggering notifications to physicians reduce understandability among users [3].
5. A free-text window triggering notifications when used should be integrated into the patient form to improve communication of other symptoms that are not present on the form or to allow patients to ask health professionals questions [3,7].
6. A software that allows the integration of patient reports into the electronic health record of the health center would be useful to improve the care team’s management of notifications [4].
7. A software that contains cancer patient education modules would assist in optimizing treatment tolerance and compliance [25].

**Figure 1:**
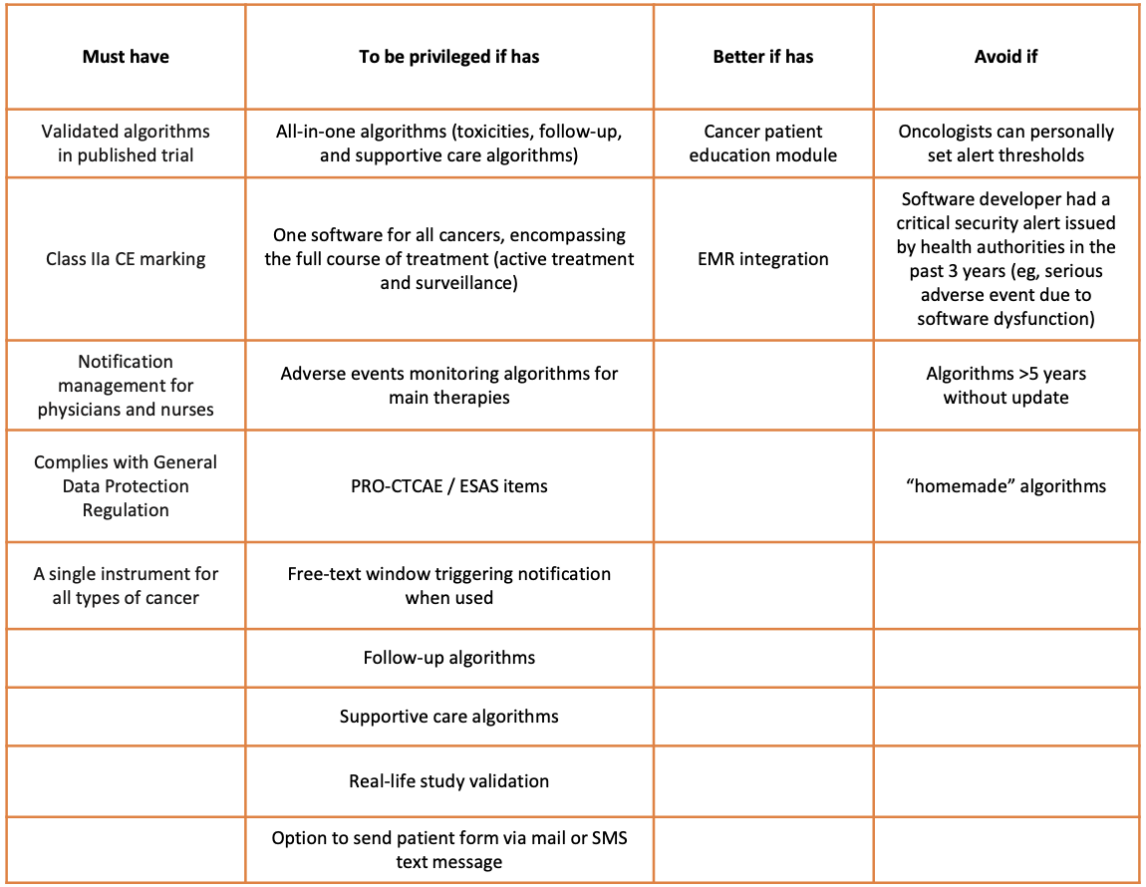
Criteria synthesis of ePRO systems for remote monitoring of patients with cancer by oncologists. EMR: electronic medical record; ePRO: electronic patient-reported outcome; ESAS: Edmonton Symptom Assessment System; PRO-CTCAE: Patient-Reported Outcomes version of the Common Terminology Criteria for Adverse Events.

These criteria are summarized in Figure 1.

As ePRO systems will become a standard of quality of care for patients with cancer, the use of these solutions will increase, but their efficacy, security, and functionality should be warranted by the software providers to give the same benefit to real-life patients to allow reimbursement for these tools by health authorities.

## Abbreviations

ePRO: electronic patient-reported outcome
ESAS: Edmonton Symptom Assessment System
NCI-CTCAE: National Cancer Institute version of the Common Terminology Criteria for Adverse Events
PRO-CTCAE: Patient-Reported Outcomes version of the Common Terminology Criteria for Adverse Events
